# The Influence of Keel Bone Damage on Welfare of Laying Hens

**DOI:** 10.3389/fvets.2018.00006

**Published:** 2018-02-28

**Authors:** Anja B. Riber, Teresa M. Casey-Trott, Mette S. Herskin

**Affiliations:** ^1^Department of Animal Science, Aarhus University, Tjele, Denmark; ^2^Animal Biosciences, University of Guelph, Guelph, ON, Canada

**Keywords:** behavior, keel bone damage, laying hen, pain, production, review, welfare

## Abstract

This article reviews current knowledge about welfare implications of keel bone damage in laying hens. As an initial part, we shortly describe the different conditions and present major risk factors as well as findings on the prevalence of the conditions. Keel bone damage is found in all types of commercial production, however with varying prevalence across systems, countries, and age of the hens. In general, the understanding of animal welfare is influenced by value-based ideas about what is important or desirable for animals to have a good life. This review covers different types of welfare indicators, including measures of affective states, basic health, and functioning as well as natural living of the birds, thereby including the typical public welfare concerns. Laying hens with keel bone fractures show marked behavioral differences in highly motivated behavior, such as perching, nest use, and locomotion, indicating reduced mobility and potentially negative affective states. It remains unclear whether keel bone fractures affect hen mortality, but there seem to be relations between the fractures and other clinical indicators of reduced welfare. Evidence of several types showing pain involvement in fractured keel bones has been published, strongly suggesting that fractures are a source of pain, at least for weeks after the occurrence. In addition, negative effects of fractures have been found in egg production. Irrespective of the underlying welfare concern, available scientific evidence showed that keel bone fractures reduce the welfare of layers in modern production systems. Due to the limited research into the welfare implications of keel bone deviation, evidence of the consequences of this condition is not as comprehensive and clear. However, indications have been found that keel bone deviations have a negative impact on the welfare of laying hens. In order to reduce the occurrence of the conditions as well as to examine how the affected birds should be treated, more research into the welfare implications of keel bone damage is needed. Research should focus on effects of genetic lines, genetic selection, housing, and nutrition for the development, prevalence, and severity of these conditions, preferably conducted as longitudinal and/or transnational studies.

## Introduction

In modern egg production, laying hens (*Gallus gallus domesticus*) face a number of clinically evident welfare problems, of which keel bone damage is among the most prevalent. As discussed by Sandilands et al. (1), selection for early sexual maturity and a continuous high egg production in commercial layer lines have led to increased bone fragility and susceptibility to fractures due to the high calcium requirement for formation of eggshells. The resulting bone weakness has mainly been associated with osteoporosis, which is a pathological condition characterized by progressive loss of structural bone throughout lay, rendering bones fragile and susceptible to fracture (2, 3). The growth of laying hen’s skeletal frame ceases at sexual maturity approximately from 16 to18 weeks of age (4). However, the ossification process of the keel bone continues until approximately 40 weeks of age (5). Hence, at 16 weeks of age as the hen begins producing eggs, several centimeters of the caudal tip of the keel remain entirely cartilaginous (6). As large amounts of calcium are required for eggshell production, starting at the onset of lay, it is possible that—for high-producing layers—the cartilaginous keel bone receives less than adequate calcium for proper ossification during the early laying period. However, at present, data are not available to support this suggestion.

**Figure 1:**
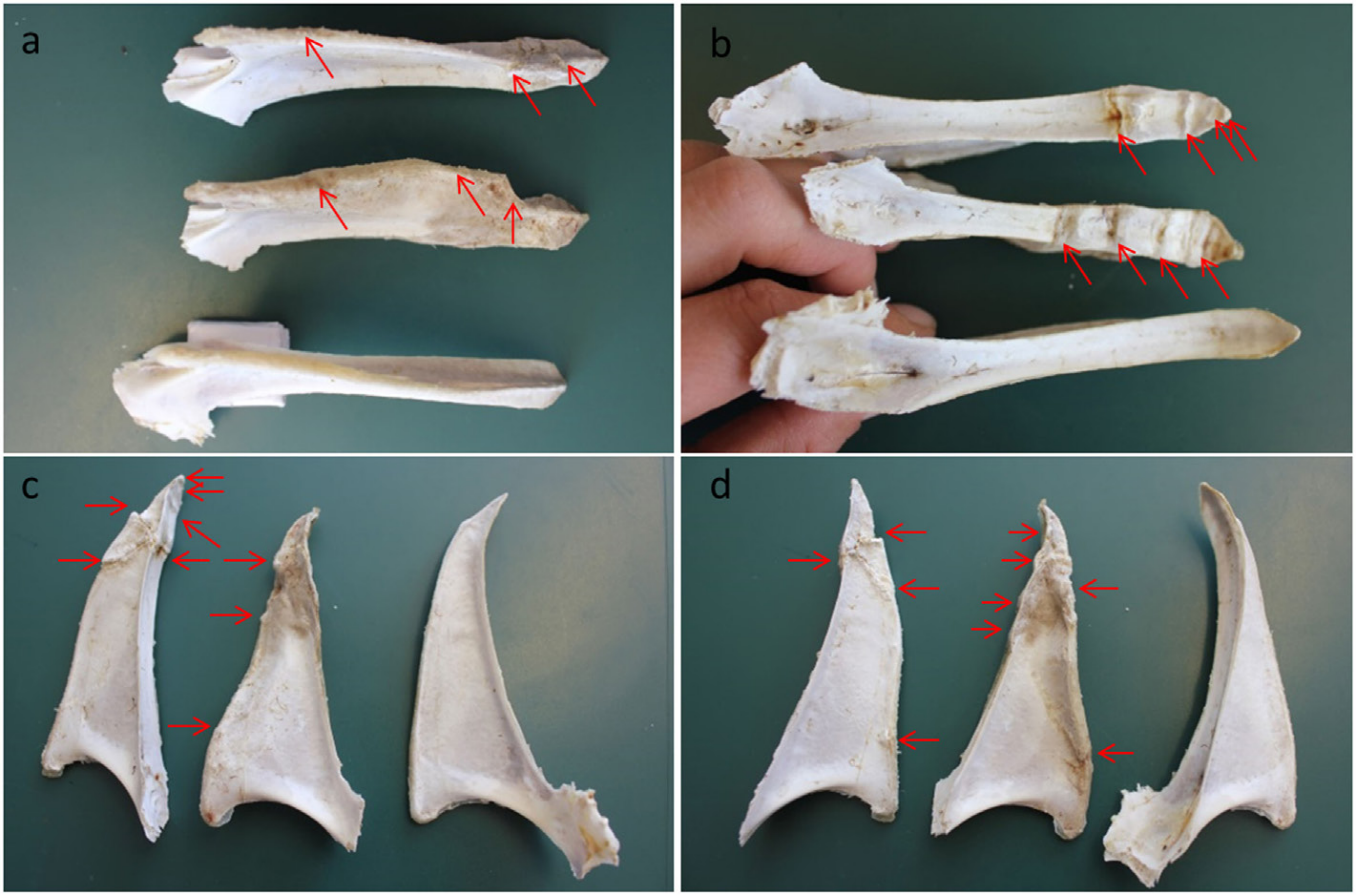
Keel bones from Danish layers aged 78 weeks with and without fractures and deviations. Three keel bones shown from different angles. The bone at the bottom in **(A,B)** and to the right in **(C,D)** is without fractures, but it has a deviation. Fractures visible on the photos are marked with red arrows on the two damaged keel bones. The different angles: **(A)** the ventral side—the tip is to the right, **(B)** the dorsal side—the tip is to the right, **(C)** the right side of the keel bones—the tip is at the top, and **(D)** the left side of the keel bones—the tip is at the top. Photos: Anja B. Riber.

The keel bone is prone to damages in terms of fractures and deviations due to the anatomical position (7, 8), especially in modern layers with small breast muscle [as discussed by Fleming et al. (7)]. Keel bone fractures are characterized by sharp bends, shearing, and/or fragmented sections of the keel bone (Figure 1). Fractures may extend from the ventral to the dorsal surface in the sagittal plane, but may also be cranial to caudal or a combination of these (9). Collision with housing structures combined with the weakened bone strength is considered the major risk factors for keel bone fractures in layers (7, 10, 11). A recent study of behavior of laying hens focused on failed landings and discussed the potential of such events for flight-related injuries (12). To model bone fractures in hens caused by collisions, Toscano et al. (13) used a drop-weight impact tester to induce keel bone fractures post mortem in layers. By employing a range of impact energies, fractures comparable to those commonly found in commercial settings were produced. These results demonstrated that impact energies of a similar order to those expected to occur during collisions in normal housing are able to produce fractures and that greater collision energies resulted in an increased likelihood of fractures and of greater fracture severity.

In recent years, high, and probably increasing [as suggested by Nasr et al. (14)], prevalence of keel bone fractures has been reported in laying hens [reports of 36–97% depending on housing system and age of the hens (7, 15–17)]. Studies on keel bone damage in meat type chickens are few, but recently Gebhardt-Henrich et al. (18) examined keel bone fractures in 45-week-old broiler breeders of the fast-growing genotype Ross 308 and the slow-growing genotype Sasso and found that Sasso birds had higher levels than Ross birds (39 vs. 15%).

Contrarily to the initial expectations, high prevalence of keel bone fractures is also found in laying hens housed in conventional and enriched cages (15, 17). Gebhardt-Henrich and Frohlich (19) showed that more new fractures occurred during the period of highest laying rates and that birds with keel bone deviations were often later diagnosed with fractured keel bones. According to the authors, the timing of the fractures occurrence did not correspond with the time when more accidents would be expected (while the hens were newly introduced to the barn).

A less often mentioned type of keel bone damage is deviation. A normal keel bone follows a straight line, but deformation may occur, leading to deviations from this line (Figure 1). These can be vertical or horizontal, showing an s-shaped appearance, bumps, or notches (8). Deviations are considered disruptions in the periosteal surface of the keel and are, thus, probably not the direct result of a fracture or impact injury as such (9). Unlike fractures, which typically happen during an isolated event, the development of deviations likely takes place over a period of time as a result of bone remodeling in response to regular loading pressures (11). Hence, long-term pressure on the keel bone during roosting is one of identified risk factor causing keel bone deviations (20–22). Pickel et al. (21) reported that hens mainly support their body weight on the keel bone while perching and that the pressure load on the keel bone is five times higher compared to the pressure load on a single foot pad. Thus, the design of perches for layers potentially affects the risk of keel bone deviations (20, 21), and increased severity of keel bone deviations has been associated with access to perches during rearing (23). Studies have reported keel bone deviations in 6–59% of laying hens aged 60–62 weeks depending on the type of production and the housing system (24, 25). In a study of broilers with access to perches, Bokkers and Koene (26) found keel bone deviations at 12 weeks of age in 19.1% of a slow-growing genotype (JA 657) and in 2.4% of a fast-growing genotype (HI-Y). Importantly, keel bone deviations can be found in intensively as well as alternatively housed laying hens, such as the organic production (27), and the described gradual, developmental process results in increased prevalence with age of the hens (28, 29). Furthermore, Gebhardt-Henrich and Frohlich (19) described how keel bone deviations often can be observed earlier in the production cycle than the more severe fractures.

The high frequencies of keel bone damage have raised the concern regarding to what extent the presence of the damage compromises the welfare of the laying hens. In this article, we aim to review the current knowledge about welfare implications of keel bone fractures and keel bone deviations, respectively, in laying hens in modern egg production. Our main focus was to examine whether the involved pathological conditions have measurable implications for the affected animals, including measures of affective states, basic health, and also the possibility of behaving naturally.

## Literature and Welfare Definition Used

The review is based on relevant scientific literature from the database “Web of Science” with “keel bone” as keyword. In addition, the review includes references from reference lists. Throughout, peer-reviewed references were prioritized, and only references written in English or German have been included.

As discussed by Robertson (30), animal welfare is a multifactorial international and domestic public-policy subject, incorporating scientific, ethical, and economic issues as well as religious, cultural, and trade considerations. Scientifically, the term “animal welfare” is often referred to as the actual state of an animal and how the animal copes with its environment (31, 32). The understanding of animal welfare, however, is influenced by value-based concerns about what is important or desirable for animals to have a good life (33, 34). These concerns can be grouped under three headings: (a) centered on the affective states of animals [as defined by Gebhardt-Henrich and Frohlich (35)] of animals, (b) centered on the ability of animals to lead reasonable natural lives, or (c) emphasizing basic animal health and production.

During recent years, the use of different types of welfare indicators for layers has been debated (36, 37), and increased use of animal-based indicators has been recommended (38). In this article, we focus on animal-based measures, covering the following categories of welfare indicators: hen behavior, physiology, clinical signs, production parameters as well as indicators of the affective states of the birds. Below, the different types of welfare indicators included are shortly introduced.

### Behavioral Measures

For decades, the behavior of farm animals, including laying hens, has been considered a key indicator of their welfare (39). Depending on the choice of behavioral test or paradigm as well as the types of observation, studies of animal behavior can be used to provide information about the time budgets of animals (40) or can be interpreted in terms of animal preferences (41) as well as the underlying motivational (42) or affective states of the animals (43, 44). Hence, across the types of concern for animal welfare, behavioral measures are considered one of the strongest types of animal welfare indicators (45, 46).

### Physiological Measures

In animal welfare science, physiological welfare indicators, including measures of immune function, have been used for decades [as reviewed in, e.g., Ref. (47)] as measures of for example animal stress (48, 49), biomarkers of disease (50), or metabolic disorders (51). However, the physiological animal welfare indicators have been criticized for their lack of specificity (37) and sensitivity [as for example for H/L-ratio as a measure of stress in laying hens (52)]. Many of the suggested physiological indicators of welfare are autonomic responses, which may indicate activity or arousal rather than being specific to poor welfare (53).

### Clinical Signs

Recordings of the clinical condition of farm animals were among the earliest welfare indicators used (54). A large focus has been on clinical measures with obvious negative consequences for animal welfare, such as mortality (55) or diseases, which especially some decades ago were considered able to stand alone as evidence for negative animal welfare [as discussed by Dawkins (53)]. In this review, we focus on effects of keel bone damage (i.e., clinical conditions) on the welfare of laying hens. Hence, effects of and interactions between keel bone damage and other clinical measures will be described in relation to the welfare of layers.

### Production Parameters

When animal welfare decreases significantly, animal fitness may be at risk (as indicated by, e.g., lower reproductive success) leading to reduced productivity in farm animals (56). However, especially for farm animals selected for high production, measures of productivity may not covary with other welfare indicators [as reviewed by Gentle (37)] and may often take a longer time to respond to a change in the welfare state of the animals than other types of indicators. Hence, when measures of animal productivity are included as welfare indicators, their validity may be lower than other types of indicators, and they cannot stand alone, especially when no effects on production are found (56, 57).

### Indicators of Affective States

Animal sentience was recognized legally in Europe in the Lisbon Treaty (58), and measures of negative as well as positive affective states are included in most welfare assessment schemes. One such example is Welfare Quality^®^ (59) where the main focus on affective states in layers is the assessment of social behavior as indicator of positive emotional states and fear of humans as well as pain during management procedures as negative affective states. As the aim of this review was to review possible welfare consequences of pathological conditions, our focus with regard to affective states has been on the negative ones, particularly pain, but evidence for other negative affective states or changes in measures of positive affective states associated with the pathological conditions in question has also been included.

Across humans and animals, the term “pain” covers an unpleasant sensory and emotional experience associated with actual or potential tissue damage or described in terms of such damage (60). Recently, it has been debated whether animal pain should be defined by listing criteria to be fulfilled (61). By using this approach, avian species fulfill all the listed criteria and are, thus, beyond any reasonable doubt, able to experience pain. The available evidence suggests that avian pain shares large similarities with mammalian pain (62–64) in terms of at least some level of cognitive component (65). In domestic fowl, nociceptors in, e.g., joints (66), beak (67), and the scaly skin on the shanks (68) have been described and characterized in terms of physiological properties such as receptive fields and thresholds. In addition, in the domestic fowl as well as in mammals, bone marrow, and growth plates are innervated, and there are nociceptors in the outer layer of the bone (2).

## Welfare Implications of Keel Bone Fractures

### Behavioral Indicators

Across Aves, the keel bone provides an anchor to which the muscles used for wing motion are attached. Hence, it may not be surprising that evidence for behavioral consequences of keel bone fractures in layers in commercial housing systems has been demonstrated. By comparing the behavior of birds with or without fractured keels, Nasr et al. (14) found that hens with keel bone fractures spent more time sleeping on the floor than hens without keel bone fractures and were less likely to access 100 cm high perches spontaneously. To date, only one study has described the behavior of individual hens prior to keel bone damage and was able to follow the behavioral changes of the birds from before development of the conditions. In the study, Gebhardt-Henrich and Frohlich (19) reared hens in an aviary system (i.e., with perches) and transferred them at 18 weeks of age to pens with elevated nest boxes, perches, and platforms. When comparing the behavior of the hens during a 10-day period before the fractured keels were identified to a similar period after fracturing, the hens stayed in the nest for a longer time during egg laying after the fractures became present. Motivational states underlying this finding were not quantified, but the authors suggested that the laying of the egg may have been difficult or even painful and, alternatively, that after the fracturing of the keels, the hens were resting more in the nests than before. Moreover, the birds were perching at night irrespective of the fractures. This finding suggests that perching is a type of behavior with limited phenotypic plasticity because of strong natural selection for escaping predation in the ancestor of the domestic fowl (i.e., the jungle fowl).

Recently, the spontaneous behavior of hens with severe versus minor or no keel bone fractures was compared for birds housed in furnished cages where the opportunity to move, fly, and access aerial perches was limited compared to floor pens or free-range systems. The fractures were divided into two categories: severe fractures involving multiple fractures and at least one complete fracture, and minor fractures involving a single “greenstick” fracture at the caudal tip of the keel bone. In the furnished cages, hens with severe keel bone fractures spent less time standing compared to hens with minor fractures or hens with non-fractured keel bones, and the standing bouts were of shorter duration. Hens with severe keel bone fractures also spent a greater percentage of time perching than hens with minor or no keel damage. Resting location differed among hens with varied keel bone status as hens with no fractures spent a greater percentage of time sleeping on the floor of the cage, whereas hens with minor fractures or severe keel damage were less often observed sleeping on the floor, subsequently spending their time resting on the perches (69). Some of the differences in sleep location among these findings, and those reported by Nasr et al. (14), are likely due to the design of the housing system. The floor pens of Nasr et al. (14) had perches at 50–150 cm off the ground and required flight to reach the roost, whereas perches in furnished cages were only 10 cm off the floor and did not require hopping or flight. Since the keel bone, as mentioned, is the site of muscle attachment for flight muscles, severe damage to the keel potentially reduces the motivation to fly to perches, unless the perches can be easily accessed. However, this explanation does not account for the reduced floor sleeping among the severely fractured birds in the study by Casey-Trott and Widowski (69); a finding which needs further study.

In a study of the behavior of free-ranging hens with access to an outdoor area, Richards et al. (70) reported a reduction in the use of pop-holes (i.e., the entrance to the outdoor area from the hen house) among hens with keel bone fractures. At 45 weeks of age, the use of pop-holes comprised 54% of non-fractured hens, 35% of minor-fractured hens, and 11% of severely fractured hens. Moreover, they considered the use of pop-holes a reasonable indicator of mobility, i.e., the observed decrease in use of the pop-holes among fractured hens indicated a reduction in mobility, especially in hens with severe fractures. In all hens, low outdoor temperatures reduced the use of pop-holes, regardless of keel bone status, but the effect was increased for hens with increasing severity of the keel bone fracture. Based on anecdotal evidence of weather-related pain in human beings, Richards et al. (70) suggested that this could be due to pain associated with healed bones and cold weather.

By use of behavioral tests introducing conflicting motivations in the birds, Nasr et al. (14) also demonstrated reductions in mobility in laying hens with fractured keel bones. Compared to birds with keel bone fractures, hens without keel bone fractures had a shorter duration for a walkway mobility test. In addition, compared to hens with keel bone fractures, birds without keel bone fractures also had a shorter latency to fly down from perches at 50 and 100 cm when presented with a feed reward at floor level. Even though more research is needed in order to fully document that the reported behavioral changes are in fact induced by the fractures, the available studies done across the relevant types of housing systems show marked behavioral differences in highly motivated types of behavior, such as perching, egg laying, and locomotion.

### Physiological Indicators

Despite the relatively long history of the use of physiological measures as animal welfare indicators, the physiological consequences of keel bone fractures are still being explored. Preliminary evidence suggests that the temperature of the area surrounding the keel is significantly higher in hens without keel bone fractures compared to hens with keel bone fractures—perhaps due to the atrophy and disuse of the breast muscle tenders (*Pectoralis minor*) or the breast muscle filets (*Pectoralis major*) (71). At present, the welfare consequences of this finding are not clear.

Although not yet explored, it is possible that damage to the keel bone may have detrimental effects on the metabolic and thermoregulatory capacities of hens. Not only is the keel bone a site of flight muscle attachment, but it is also the site of muscle attachment for respiratory motions involved in inhalation and exhalation (72). Since avian species lack a muscular diaphragm, they rely on the oscillation of the keel bone and ribs to drive inhalation and exhalation (72–74). If the keel bone is severely damaged, the involvement of the keel in respiration may be reduced due to pain or physical restriction of motion, potentially influencing the metabolic or thermoregulatory capacity of the birds. Hence, some physiological effects of keel bone fractures have been identified, and other potential effects still need to be clarified. Common for both types is that their consequences in terms of animal welfare are unknown.

### Clinical Indicators

The presence of keel bone fractures is in itself a clinical parameter, which is an indicator of reduced welfare (2). Regarding hen mortality, not much is known about possible relations to keel bone fractures. Recently, Kajlich et al. (75) examined welfare implications of lesions observed post mortem on hens that died or were culled from three non-cage US farms but did not report the occurrence of keel bone fractures. Nasr et al. (14) showed no data, but stated that hens with fractured keel bones show high survival rate. In an older study, McCoy et al. (76) showed associations between osteoporosis and hen mortality in caged layers but did not present data on keel bone fractures. Furthermore, Heerkens et al. (24), performing an on-farm study, nor Gebhardt-Henrich and Frohlich (19), performing an experimental study, found associations between keel bone fractures and mortality.

Even though Nasr et al. (14, 77) did not find differences in the body weight of hens with or without keel bone fractures, the fractured birds ate more feed and consumed more water than the control birds when comparisons were made after diagnosing the fractures (77). The presence of keel bone fractures has, however, not only been associated with changes in classical measures of body condition and intake of feed and water. More recently, Gebhardt-Henrich and Frohlich (19) found a relation between the occurrence of bumble foot and the presence of keel bone fractures at the end of lay and suggested that hens with bumble foot may be more prone to slipping or falling from perches. Likewise, keel bone damage has been found to be linked with poor feather coverage (Riber and Hinrichsen, accepted). The causal relationship has not yet been clarified, but it is suggested that fearfulness increases in flocks experiencing high levels of injurious pecking, resulting in flighty birds that have a higher risk of both keel bone fractures due to more uncontrolled landings and take-offs and keel bone deviations as fearful birds may spend more time perching.

Hence, at present, relatively little is known about how keel bone fractures affect or interact with other clinical parameters, but the presence of fractures is an indicator of reduced welfare, and there seem to be relations between the fractures and other clinical indicators of reduced welfare such as bumble foot.

### Indicators of Affective State

In humans and mammals, bone fractures are in general acutely painful (78–81). Subsequent influx of inflammatory mediators to the site of fracture leads to further local stimulation of nociceptors which, when studied in humans and rodent models, may trigger peripheral as well as central sensitization manifested as hyperalgesia and/or prolonged pain even during or after healing (81). Typically, these effects are worse, and healing takes longer if a fracture site is mobile during repair.

To date, the sensory innervation of avian keel bones has not been studied, but similarities in bone physiology and fracture healing in mammals and birds strongly suggest that the keel bone is densely innervated by sensory afferent fibers [as discussed by Webster (3) and Nasr et al. (14)]. In addition, as mentioned above, behavioral changes can be used as signs of affective states such as pain (43, 44). Some of the listed behavioral effects of keel bone fractures, such as increased time spent in the nest at egg laying, reduced mobility, or reluctance to perform the natural behavior of perching, may indicate that the presence of the fractures is associated with negative affective states such as discomfort and/or pain. Based on this knowledge, several studies have assumed that the fractures are painful (82). In addition, the finding by Nasr et al. (14) showing that hens with keel bone fractures had an increased latency to fly down from perches of 100 and 150 cm compared to hens without keel bone fractures adds to suggestion of the presence of negative affective states such as pain. As argued by Weary et al. (44), such data strengthen the inference regarding a negative emotional state because these were based on a testable prediction involving the creation of a test situation encouraging a specific behavioral response. According to this line of argumentation, the authors further added to the evidence suggesting involvement of pain by demonstrated that administration of the analgesic butorphanol reduced the latency to fly down only in the fractured hens. In addition, a follow-up study then demonstrated a conditioned place preference after administration of butorphanol only in hens with keel bone fractures, not in control hens (83). The finding of a preference for the location where the effects of butorphanol were experienced indicates that the analgesic effect of the drug was rewarding for hens from the fractured group and had no effect on hens with non-fractured keel bones, suggesting a positive affective state induced by the analgesic drug.

As mentioned, the keel bone of hens is vulnerable to movement caused by flight or perching (70). Hence, Nasr et al. (14) suggested that such normal types of hen behavior are likely to cause disruption of any acute keel bone fracture and to generate nociceptive activity. This is particularly a problem when a hen must move to reach food, water, and a nest box (2). However, at present, it is not known how the pain develops during the period after the bones are fractured, or how long these consequences in terms of pain may persist in layers with fractured keel bones. The experiments described above involving conditioned place preferences involved birds with fractures of at least three weeks, suggesting that fractures are a source of pain—at least for weeks after the trauma occurs.

### Production Parameters

In an experimental study, Nasr et al. (14) investigated the effects of keel bone fractures on egg production parameters. Hens with keel bone fractures both laid fewer eggs and eggs with a lower eggshell weight. In addition, there was a tendency for hens with keel bone fractures to lay lighter eggs with a smaller surface area. In an experimental follow-up study, Nasr et al. (77) again found that hens with keel bone fractures laid fewer and lighter eggs compared to hens without keel bone fractures, thus supporting their previous results. Recently, Candelotto et al. (84) also found evidence for an association between lowered egg quality and susceptibility to keel bone fractures.

Rufener et al. (85) presented data from a commercial aviary system, showing that hens with fractured keel bones tended to have a reduced laying performance compared with hens with intact keels, but that the former laid heavier eggs. In their longitudinal study, Gebhardt-Henrich and Frohlich (19) found that an early initiation of egg production was positively associated with the presence of keel bone fractures at the end of the study, whereas no associations were found between the total number of eggs produced and the presence of keel bone fractures. Similarly, Heerkens et al. (24) did not find associations between keel bone fractures and egg production in an on-farm study of 47 flocks of laying hens housed in aviaries. Likewise, Gebhardt-Henrich and Frohlich (19) did not find differences in the rate of egg production between a 28-day prefracture period and a similar period after the diagnosis of the keel bone fractures.

Thus, so far, effects of keel bone fractures on measures of egg production have been found in some experimental studies but have not been verified in on-farm studies. Despite the discrepancy in the findings of relations between keel bone fractures and measures of egg productivity, Toscano (86) stated that keel bone fractures represent decreased profitability resulting from splintered bone in breast meat reducing carcass value in countries where laying hens are used for human consumption.

## Welfare Implications of Keel Bone Deviations

Compared to the evidence presented above regarding the effects of fractured keel bones of layers on measures of animal welfare, much less is known about the consequences of keel bone deviations in these birds; a subject which, to date, is largely unexplored. Since keel bone deviations are frequently found in conjunction with keel bone fractures, the majority of the available studies have categorized these together, and it is thus, in many cases, difficult to infer only the effect of the deviations on animal welfare due to the experimental design of the studies. Below, the available knowledge about consequences of keel bone deviations in terms of animal welfare is reviewed. For all the welfare indicators reviewed, knowledge on how this pathological condition affects animal welfare is limited, calling for future studies on this topic.

### Behavioral Indicators

Severe keel bone deviations have been suggested to impair motion and rest due to the attachment of the breast muscles to the keel bone (20, 87).

### Clinical Indicators

As for the keel bone fractures, the presence of deviating keel bones is a clinical parameter in its own, which may indicate reduced welfare (2). However, even less is known about how the presence of keel bone deviations affects or interacts with other clinical parameters.

Recently, Kajlich et al. (75) examined welfare implications observed in post mortem examinations of hens that died or were culled from three non-cage US farms and observed keel bone deviations in over 40% of the dead birds, especially during mid lay. Hence, keel bone deviations were among the most common clinical findings in the dead hens.

As mentioned, Gebhardt-Henrich and Frohlich (19) suggested that birds with deviations have higher risk of fracturing their keel bones. This has been supported by Harlander-Matauschek et al. (10) stating that keel bone deviations may lead to unequal bone loading during wing-flapping and concentration of strain energy in ways that increase the risk of fractures. Furthermore, Fleming et al. (7) found that hens with normal keel bones had larger breaking strength on humerus and tibiotarsus than hens with deviating keels.

### Indicators of Affective States

At present, no studies have focused on effects of keel bone deviations on the affective states of layers. It can, however, not be excluded that the developmental process of the deviations or the period of time where the birds have to live with these conditions is associated with changes in the affective states of the birds, especially because of the effects of keel bone damage on highly motivated natural behavior, such as perching and use of elevated nest boxes (14).

### Physiological Indicators

Separating out the effects of keel bone deviations from keel bone fractures on physiological indicators of animal welfare is largely unexplored.

### Production Parameters

To the best of our knowledge, to date, only one study has examined effects of keel bone deviations on measures of egg production. In an on-farm study of 47 flocks of laying hens housed in aviaries, Heerkens et al. (24) found that the percentage of second-quality eggs was associated with the percentage of hens with a keel bone deviation, but the direction of the association is not clearly described.

## Discussion

The present review is based on recent findings of a high prevalence of keel bone damage in commercial laying hens in caged as well as non-caged production systems (7, 15–17, 24, 75, 88). We have reviewed the current knowledge about welfare implications of keel bone fractures and deviations in laying hens (Table 1). As an initial part, the different conditions were shortly described, and major risk factors were presented together with findings on the prevalence of the conditions. Our main focus has been to examine whether keel bone fractures and deviations have measurable consequences for the affected animals in an evidence-based evaluation of layer welfare. Below, the reported findings are discussed in terms of evidence across different types of welfare indicators. Also, possible interactions between the pathological conditions and factors, such as age of the hens and housing system, are considered.

**Table 1:** A simplified summary of previous research reporting main findings of welfare indicators of keel bone damage reviewed in this article.

	Indicator				
Reference	Behavioral	Physiological	Clinical	Affective state	Production
(14)	↑ Sleep on floor ↓ Use of perches ↑ Latency fly off perches ↑ Duration walkway test	↓ Temperature around keel ↓ Keel bone strength	No difference BW	N/A	↓ Eggs laid ↓ Eggshell weight ↑ Lighter egg weight ↓ Egg surface area
(71)	N/A	N/A	N/A	↓ Latency fly off perch after analgesics	N/A
(70)	↓ Use of pop-holes	N/A	N/A	N/A	N/A
(82)	N/A	↓ Tibia ash content ↓ Tibia breaking strength	No difference BW No difference girth or wing:girth ratio ↓ Feather coverage	N/A	N/A
(77)	N/A	N/A	N/A	CPP formed with analgesics	N/A
(83)	N/A	↓ Keel bone strength	No difference BW No difference in egg deformation, blood spots or calcification	N/A	↑ Feed intake ↑ Water intake ↓ Eggs laid ↓ Egg weight
(13)	N/A	↓ BMD keel surface ↓ peak load failure tibia	N/A	N/A	N/A
(19)	↑ Time in nest boxes after keel fracture No difference night perch use	N/A	↑ Incidence of bumble foot	N/A	Earlier first egg appearance No difference total egg number
(86)	N/A	N/A	N/A	N/A	↑ Bone splinters in breast muscle
(69)	↓ Time standing ↓ Standing bouts ↑ Time perching ↑ Rest on perches	N/A	N/A	N/A	N/A
(85)	N/A	N/A	N/A	N/A	↓ Laying performance ↑ Egg weight
(24)	N/A	N/A	N/A	N/A	↓ Egg production ↓ 2nd quality eggs
(98)	No difference in balance measures while perching No difference latency to fly off perch	N/A	N/A	N/A	N/A
(99)	N/A	↓ Keel calcium content ↓ Tibia shear strength	↑ Body mass	N/A	N/A
(84)	N/A	N/A	N/A	N/A	↓ Eggshell strength
(100)	N/A	N/A	↓ Poor plumage condition	N/A	N/A

*The text lists measures where birds with keel bone fractures differed significantly from the non-fractured birds as well as measures where no differences were found*.

*N/A, not available; BW, body weight; CPP, conditioned place preference; BMD, bone mass density*.

### Evidence for Welfare Implications of Keel Bone Damage

Keel bone fractures have been shown to affect most of the treated welfare indicators, i.e., keel bone fractures prevent the birds from performing motivated behavior, are painful, and seem to have negative effects on egg production. The conclusion that keel bone fractures have negative consequences for the welfare of laying hens has indeed been suggested by several authors (14, 19, 77, 83). In 2010 and again in 2013, the Farm Animal Welfare Council in the UK expressed strong concerns about the negative impact of bone fractures on the welfare of laying hens (2, 89). They stated that keel bone damage represents one of the greatest welfare problems facing the laying hen industry.

In this review, we included not only fractures but also keel bone deviations. As these are frequently found in conjunction with fractures of the keel bone, the majority of the available studies have categorized these conditions together, and it is thus, in many cases, difficult to infer only the effect of the deviations on animal welfare. Hence, the welfare consequences of keel bone deviations in laying hens remain largely unclear, but it has been suggested that they have negative effects on welfare in terms of causing increased risk of fractures and impaired movement and rest. Kajlich et al. (75) concluded that keel bone damage, involving deviations and fractures, is one of the most serious welfare problems in commercial non-cage egg production because of its high prevalence, the pain involved, and the reduced mobility of the birds. As emphasized by Harlander-Matauschek et al. (10), more research should be addressed to the relationship between keel bone deviations and keel bone fractures. Increased knowledge on this topic will be of value in the evaluation of the welfare implications of keel bone deviations in laying hens.

The two treated types of keel bone damage are widespread; they can be found in all housing systems and in all strains of commercial laying hens (20, 25, 90) and under different types of management. Multiple factors are involved in the causation of the conditions, complicating future solutions. So far, studies on the welfare consequences of these conditions have mainly been cohort studies (91) where the experimental birds have been included based on a diagnosis with the condition under study and then compared to matched controls. However, it cannot be excluded that findings of simple differences in for example behavior from such studies are due to differences between the birds, which were also present before the occurrence of the condition, or even that the behavioral findings could be potential risk factors for the development of the condition. If studies have involved longitudinal data collection, i.e., where the study populations are followed before, during, and after the appearance of the condition [such as (19)], or involved interventions [such as the use of analgesic drugs by Sherwin et al. (83)], the evidence is stronger. Including a description of the severity and the location of the fracture(s) on the keel bone in future studies is also of utmost importance as more evidence is needed to describe whether or not all types of keel bone damage are equally painful. In this review, we have tried to take the experimental designs of the reported studies into account.

### Age of Hens and Housing System Affect the Welfare Implications of Keel Bone Damage

When assessing the welfare consequences of keel bone damage, the age of the hens is an important factor. Some of the conditions are more likely to occur at specific ages and/or to accumulate with age. Several studies have shown that the prevalence of keel bone fractures increases throughout the laying period (6, 25, 70, 92). In addition, the prolonged consequences in terms of pain suggested by, e.g., the work of Nasr et al. (83) as well as the potential for these fractures to lead to conditions known from humans and non-human mammalian model species [such as central sensitization, manifested as hyperalgesia and prolonged pain during or even after healing (81)] mean that the consequences in terms of pain may be persistent and even become sensitized over time due to, e.g., disruption of the fractures [as suggested by Nasr et al. (14)]. Thus, a hen with a keel bone fracturing early in the production period may be subject to poor welfare during a substantial part of her lifespan. In addition, Richards et al. (70) found that the severity of keel bone fractures increased with age. Considering the extent and severity of keel bone fractures at the end of lay, handling and transportation of end-of-lay hens is of great concern, but so far, research into the welfare consequences of keel bone damage during poultry transport does not exist.

As touched upon in the introduction, the prevalence, type, and severity of keel bone damage depend to some extent on the housing system. Among the major risk factors seems to be whether it is a cage or non-cage system, and for the latter, whether it has a single tier or multi-tiers. Keel bone damage seems to be more frequent in non-cage systems than in cage systems (15–17) and more in multitier systems than in single-tier systems (8, 25). Toscano et al. (93) stated that the move toward non-cage systems in Europe and Northern America poses a challenge for solving the problems with keel bone damage in the future.

In addition, there is a general consensus that perches/tiers have a causal role for the development of some of the types of damages to keel bones (10, 11, 21, 94). Nevertheless, removing perches and tiers will not be a useful solution, as this action would cause other welfare problems for the laying hens. Hens are highly motivated to perch for roosting and display signs of unrest and frustration if access to perches is denied (95, 96). Thus, perches/tiers are important resources for laying hens, providing an essential place for roosting, which is a highly valued activity and part of the natural behavior of a laying hen. However, recent studies have shown that proper design of the housing environment, including the perches, may alleviate the prevalence of keel bone damage. Pickel et al. (21) found that the perch design affected the pressure load against the keel bone and foot when the hens were roosting. This was supported by Stratmann et al. (11) who found that the prevalence of keel bone fractures can be reduced by provision of soft perches. Also, Stratmann et al. (94) found more controlled movements, fewer falls and fewer collisions when ramps were provided between the tiers in a multi-tiered aviary system. As a result, fewer fractured keel bones were found at 60 weeks of age, but at 66 weeks of age the difference had disappeared.

Thus, interactions between housing systems and the clinically evident welfare problems reviewed in this report exist. The prevalence of the conditions varies among different housing systems, and the negative consequences of having to live with the conditions in the different housing systems are likely to differ, too. For example, the reduced mobility caused by keel bone fractures will have a greater impact on hens in multi-tier systems, where vital resources (feed, water, and nest boxes) are found on different tiers, than on caged hens living in a highly restricted area. Despite the risks from perches/tiers for the development of keel bone damage, these environmental structures are considered a behavioral need for laying hens and cannot be removed from housing systems for layers without reducing the welfare of all birds in a flock.

### Weighing of the Different Welfare Indicators?

In this review, we have covered several types of welfare indicators, including measures of affective states, basic health and functioning, and natural living. We have, however, not been weighing the different measures against each other; a process which is not straightforward [as discussed by Gentle (37)] and is often done by, e.g., use of expert panels [as discussed by Webster (97) for laying hens]. Nevertheless, as the vast majority of the results presented above—across the different types of indicators—point in the same direction, the weighing of them has not been considered central.

It could be argued that the presence of the keel bone damage can stand alone as a sign of reduced welfare for the layers [as discussed by Keeling et al., (53)]. In this review, however, we have added the available information about other aspects of the welfare of hens, such as their affective states and their possibility to behave naturally, in order to strengthen the conclusions and to approach the raised questions from as many angles as possible. The majority of the evidence provided comes from the behavioral welfare indicators (which are often considered key indicators of animal welfare (46)) showing effects on time budgets of the birds, their preferences and underlying affective states. For the vast majority of the reported studies, the behavioral results point in the same direction, namely the fact that especially keel bone fractures, and to a lesser extent keel bone deviations (primarily due to lack of studies or lack of precise definitions in the studies), have negative consequences for the welfare of laying hens.

It is well known that not all measures of animal welfare have comparable sensitivity [as discussed by Mendl (57)]. One typical example of this, especially in high-producing farm animals such as layers, is the measures of productivity, which may often take longer time to respond to a change in the welfare state of the animals than other types of welfare indicators (37). The lack of consistency between experimental and on-farm studies in the reported findings regarding relations between keel bone fractures and egg production may be a reflection of this. However, it is important to stress that when measures of animal productivity are included as welfare indicators, they cannot stand alone, especially when no effects on production are found (56, 57). Hence, the lack of evidence for effects on keel bone deviations on laying hen productivity cannot be used to document that the welfare of the hens is not affected by these conditions.

## Conclusion

Irrespective of the underlying welfare concern, the available scientific evidence shows that keel bone fractures reduce the welfare of layers in modern production systems. Due to the limited research into the welfare consequences of keel bone deviations, evidence of the consequences of these conditions is not as comprehensive and clear. However, indications have been found that keel bone deviations have negative impact on the welfare of laying hens. In order to be able to reduce the occurrence of the conditions as well as to examine how the affected birds should be treated, more research into the welfare consequences of keel bone damage is needed. This research should focus on effects of genetic lines, genetic selection, housing and nutrition for the development, and prevalence of these conditions, preferably conducted as longitudinal and/or transnational studies.

## Author Contributions

AR conceived the idea of the review. The manuscript was prepared and edited by all authors.

## Conflict of Interest Statement

The authors declare that the work was conducted in the absence of any commercial or financial relationships that could be construed as a potential conflict of interest.
